# Editorial: Bispecific antibodies and their conjugates in solid tumors and hematological malignancies

**DOI:** 10.3389/fimmu.2025.1752360

**Published:** 2025-12-09

**Authors:** Mirosława Püsküllüoğlu, Renata Pacholczak-Madej

**Affiliations:** 1 Department of Clinical Oncology, Maria Skłodowska-Curie National Research Institute of Oncology, Kraków, Poland; 2 Department of Gynecological Oncology, Maria Skłodowska-Curie National Research Institute of Oncology, Kraków, Poland; 3 Department of Anatomy, Jagiellonian University Medical College, Kraków, Poland

**Keywords:** bispecific antibodies, antibody-drug conjugates, hematological malignancies, solid tumors, emerging antibody technologies

Bispecific antibodies and related multispecific formats have evolved remarkably quickly from conceptual immuno-oncology tools to routine components of therapeutic algorithms in hematology and, increasingly, in solid tumors (1–3). This Research Topic was conceived to capture that transition across the full translational continuum: from antibody engineering and mechanistic studies in complex preclinical models, through early and late-phase clinical data, to systematic syntheses and critical evaluation of real-world and trial-based evidence. The articles collected here illustrate how rapidly the field is evolving, but also how heterogeneous the underlying biology, clinical development strategies and toxicity profiles remain.

Several contributions focus on acute leukemia, where bispecific T cell engagers were first clinically established. Cao et al. summarize the most recent bispecific antibody data presented at the 66th American Society of Hematology meeting, encompassing both acute lymphoblastic leukemia (ALL) and acute myeloid leukemia, as well as combinations with chemotherapy and other targeted agents. Their conference-based overview highlights not only consistently high measurable residual disease (MRD) negativity rates in relapsed/refractory B-ALL but also the diversification of targets and platforms entering clinical testing, as well as the operational challenges of delivering these agents outside highly specialized centers.

Blinatumomab, as the prototypical Cluster of differentiation (CD)19×CD3 T cell engager, is examined in depth in two complementary articles focused on pediatric B-ALL. Cheng and Liu provide a structured review of clinical trials of blinatumomab in children, emphasizing how disease burden, endogenous T cell competence, CD19 antigen modulation and lineage switch influence efficacy and relapse patterns, and how cytokine release syndrome and neurotoxicity can be anticipated and managed in this age group. In parallel, Zhang et al. report a multicenter pediatric cohort in which blinatumomab was used both as preemptive therapy in MRD-positive or chemotherapy-delayed patients and as reinduction in relapsed/refractory disease. They document high MRD-eradication rates in chemotherapy-delayed and MRD-positive cohorts, response rates in frank relapse comparable to those seen in registrational trials, and identify adverse cytogenetics, CD19 loss and Breakpoint cluster region–Abelson 1 fusion positivity as predictors of inferior response. Together, these two articles illustrate how a single bispecific antibody can be integrated at different decision points along the paediatric ALL treatment trajectory, and how careful characterization of response determinants can guide patient selection and sequencing with transplantation or Chimeric antigen receptor T cell therapy.

In multiple myeloma, Li et al. present a systematic review and meta-analysis of teclistamab, a B cell maturation antigen×CD3 bispecific antibody, across clinical trials and real-world cohorts. Their synthesis confirms a survival advantage over existing regimens in relapsed/refractory disease, with robust response rates and deep remissions, while also demonstrating that patients treated outside of trials tend to have somewhat lower survival outcomes, likely reflecting shorter follow-up and higher baseline risk. Subgroup analyses suggest that combination regimens can further enhance response depth, at the cost of added toxicity. At the opposite end of the evidence spectrum, Chu et al. describe the successful use of the CD20×CD3 bispecific antibody glofitamab as salvage therapy in a patient with primary refractory diffuse large B cell lymphoma/high-grade B cell lymphoma-MYC proto-oncogene/B cell lymphoma 2 transformed from follicular lymphoma and resistant to modern chemoimmunotherapy. This carefully documented case illustrates how bispecific antibodies can provide meaningful disease control even in highly adverse biological subsets and argues for their timely consideration in transformed and double-hit lymphomas.

Beyond hematologic malignancies, several articles address dual-target and multispecific strategies in lung cancer and other solid tumors. Zhang et al. conducted a systematic review and meta-analysis of phase III randomized trials of dual-target immunotherapies in advanced non-small cell lung cancer, including bispecific antibodies and other dual-pathways. Their analysis shows improvements in progression-free survival and objective response compared with conventional regimens, but no clear overall survival benefit to date, and a consistent increase in treatment-related toxicity, particularly with Epidermal growth factor receptor/MET proto-oncogene-directed strategies. These findings underscore the need for better biomarker-driven patient selection and rational toxicity mitigation when multiple signaling or immune pathways are targeted simultaneously. Complementing this, Chen et al. provide a focused review of bispecific antibodies in lung cancer, describing the structural diversity of these agents, the range of antigen combinations under clinical investigation, and the mechanistic rationale for engaging immune effector cells or co-targeting oncogenic drivers. The accompanying correction, in which the authors amend the global lung cancer incidence figure, serves as a reminder that the rapid pace of progress must be matched by equal rigor in epidemiological and contextual reporting.

Three contributions illustrate how antibody engineering is being used to refine the balance between potency, selectivity and developability of next-generation molecules. Lin et al. characterize JS207, a bispecific antibody targeting Programmed cell death protein 1 and Vascular endothelial growth factor A, designed to deliver localized dual checkpoint and anti-angiogenic blockade. They show preserved binding to both targets, effective T cell activation, favorable internalization properties and encouraging antitumor activity in preclinical models, together with enhanced thermal stability relevant for manufacturing and shelf life. Ma et al. develop B7 homolog 6–targeted bispecific antibodies combined with Interleukin-15 receptor alpha chain sushi fusion to co-engage T and Natural killer cells against solid tumors resistant to chemotherapy. In xenograft models, they demonstrate dose-dependent tumor suppression and synergistic effects of combining two B7-H6–directed formats, supporting the concept that simultaneous recruitment of distinct effector compartments may overcome resistance in heavily pretreated disease. Löffler et al. introduce an engineered Fab–Fab-engineered immunoglobulin (eFab-eIg) trispecific platform that incorporates one classical Fab and two eFab moieties to achieve co-targeting of the human epidermal growth factor receptor 2 (HER2)/human epidermal growth factor receptor 3 (HER3) with CD3 engagement. Using two-dimensional and three-dimensional cancer models, they show that this modular architecture enables potent T cell retargeting against HER2/HER3-expressing tumor cells, while illustrating how stoichiometry and spatial arrangement can be exploited to tune activity and potentially reduce off-tumor effects.

This Research Topic shows bispecific and other multispecific antibodies that move from hematologic malignancies into lung and other solid tumors, including heavily pretreated, high-risk patients. They enable MRD-focused strategies, options for such disease and precision use guided by immunophenotype and genetics, but at the price of immune toxicities, serious infections and infusions that require close monitoring (4, 5). Translational work on cytokine-fusion, trispecific and other advanced formats shows how they remodel the tumor microenvironment, mobilize effector cells and counter immune escape and other resistance. Meta-analyses, real-world cohorts and smaller clinical series define priorities: biomarker-based target selection, integration with cellular therapies and radiation therapy, and long-term safety, sequencing, and survivorship study. Built to bridge diseases and disciplines, this 2024–2025 snapshot spans diverse cancers and study stages and aims to inform the next generation of bispecific and multispecific therapies.
